# Integrated Pathway Clusters with Coherent Biological Themes for Target Prioritisation

**DOI:** 10.1371/journal.pone.0099030

**Published:** 2014-06-11

**Authors:** Yi-An Chen, Lokesh P. Tripathi, Benoit H. Dessailly, Johan Nyström-Persson, Shandar Ahmad, Kenji Mizuguchi

**Affiliations:** National Institute of Biomedical Innovation, Ibaraki, Osaka, Japan; Georgia Institute of Technology, United States of America

## Abstract

Prioritising candidate genes for further experimental characterisation is an essential, yet challenging task in biomedical research. One way of achieving this goal is to identify specific biological themes that are enriched within the gene set of interest to obtain insights into the biological phenomena under study. Biological pathway data have been particularly useful in identifying functional associations of genes and/or gene sets. However, biological pathway information as compiled in varied repositories often differs in scope and content, preventing a more effective and comprehensive characterisation of gene sets. Here we describe a new approach to constructing biologically coherent gene sets from pathway data in major public repositories and employing them for functional analysis of large gene sets. We first revealed significant overlaps in gene content between different pathways and then defined a clustering method based on the shared gene content and the similarity of gene overlap patterns. We established the biological relevance of the constructed pathway clusters using independent quantitative measures and we finally demonstrated the effectiveness of the constructed pathway clusters in comparative functional enrichment analysis of gene sets associated with diverse human diseases gathered from the literature. The pathway clusters and gene mappings have been integrated into the TargetMine data warehouse and are likely to provide a concise, manageable and biologically relevant means of functional analysis of gene sets and to facilitate candidate gene prioritisation.

## Introduction

There has been an exponential increase in the amount and complexity of biological data. Extracting meaningful biological insights from this vast array of data via functional analysis of the large resultant gene sets and to prioritise genes and gene sets for further experimental characterisation is a formidable challenge. Gene-set-functional-enrichment (GSFE) relies on a statistical analysis of the relative abundance of biological themes associated with a given gene set and identifies themes (and associated genes) that are overrepresented and therefore, likely to be more relevant to the biological conditions under study.

It is increasingly evident that gene and proteins do not function alone, but rather as a part of complex pathways where they interact with various biomolecules (such as proteins, nucleic acids and metabolites). Therefore, an accurate representation of biological pathway information is essential to understand the biological relevance of genes and proteins within specific biological contexts. An ever growing number of pathway databases, thereby, constitute an increasingly important component of any computational framework for the functional annotation of genes and proteins. However, the available pathway resources often differ widely in scope and content, which severely hampers a unified analysis and interpretation of high-throughput biological data using diverse pathway repositories [1]–[3]. In the absence of reasonable compatibility, a unified representation of gene function by leveraging the biological information stored in various pathway repositories remains a non-trivial task.

**Figure 1:**
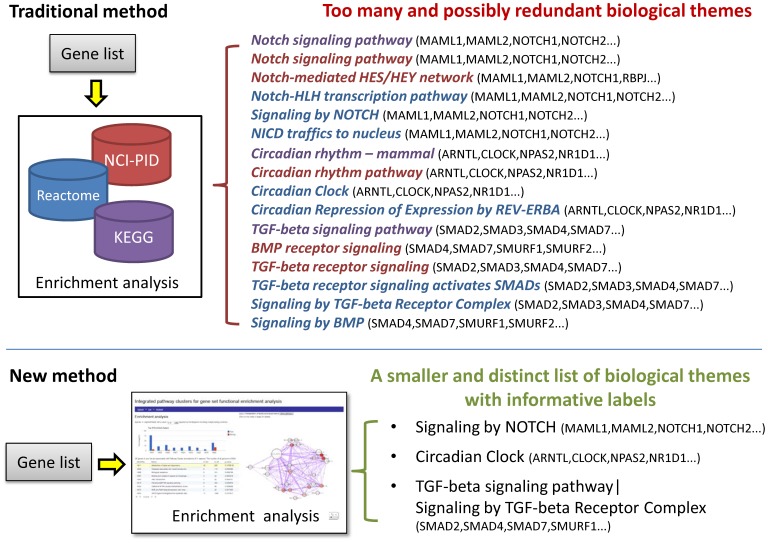
Benefits of using an integrated pathway repository for GSFE analysis.

Integration of pathway repositories offers significantly attractive benefits in terms of more extensive and robust functional annotations, which in turn will contribute to a better understanding of gene function and regulation in complex biological systems. Furthermore, it also lends itself to providing a more concise and relatively discrete representation of enriched biological themes in combined GSFE studies (Figure 1). In recognition of these benefits, several efforts have been initiated to gather and integrate biological data, including pathway information from various biological databases. The DAVID gene functional classification tool employs a heuristic approach to grouping genes into modules based on similarities in the biological annotations [4]. IPAD defines inter-associations between pathways, disease, drugs and organ specificity based on the overlapping gene associations [5]. IntPath examines overlaps between genes, gene pairs and pathway names to integrate pathways within and across various databases [6]. PathwayAPI attempts to standardise the representation of genes and gene-gene relationships across pathways and merges them to infer more fortified pathway representations [1]. Pathway Distiller employs a holistic approach where pathways are consolidated into clusters either based on shared genes, gene ontology associations and protein-protein interactions (PPIs) or based on their associations (enriched and/or non-enriched) with specific gene sets under study [7]. Most of these tools, however, provide a standalone web interface and have not been integrated into a more general data-mining platform. Such a platform is often essential for prioritising genes for further characterisation in drug discovery and other applications.

Here we describe a new approach to integrating pathway data primarily for target prioritisation. While our method for pathway integration is simple and straightforward, the main novelty lies in its tight integration into the TargetMine data warehouse system [8]. We chose to combine data from three pathway repositories, KEGG [9], Reactome [10] and NCI-Nature curated PID [11]. These three are among the largest and most widely used curated pathway repositories and they employ different approaches to curating and compiling pathway information. For instance, the KEGG pathway repository consists of curated reference pathway maps, which are then mapped to genes within different organisms based on orthologous associations. Reactome compiles expert-curated molecular reactions associated with different biological processes, which are assembled into a biomolecular network to form pathways. NCI PID compiles expert-reviewed molecular interaction data from NCI-Nature curated data, BioCarta and Reactome into biomolecular pathways.

We will first show how the various pathways can be clusters based on shared gene content, on the premise that significant overlaps in gene content between the pathways should reflect overall functional congruity between them. This notion will be confirmed by the biological relevance of the integrated pathway clusters using semantic similarities between Gene Ontology (GO) biological process terms [12] (hereafter referred to as GO terms) annotated to the genes within each pathway. We will further demonstrate the usefulness of pathway clustering based on comparative GSFE analysis on diverse gene sets associated with pathogenesis, inflammatory responses and human diseases, gathered from the literature. A dedicated user interface connects the pathway clusters and gene mappings to TargetMine, for target prioritisation and early-stage drug discovery [8].

## Results and Discussion

**Figure 2:**
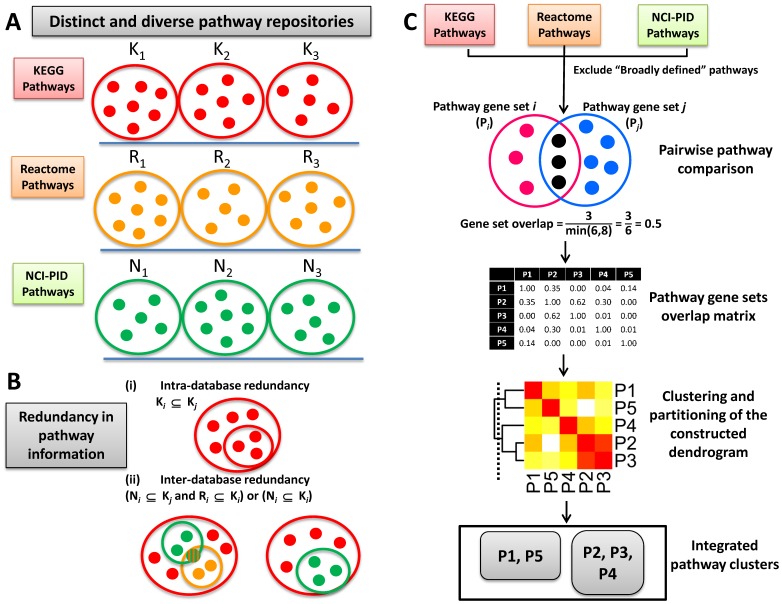
Overcoming the challenges encountered in integrating diverse pathway data. A) Pathway data are compiled in varied repositories, which differ appreciably in scope and content and B) there exists significant redundancy within pathway definitions among different databases. C) An outline of our approach to integrating pathway data from KEGG, Reactome and NCI-PID databases.

By integrating pathway data from KEGG, Reactome and NCI, we created Integrated Pathway Clusters (IPCs) for three organisms, *Homo sapiens* (human), *Mus musculus* (mouse) and *Rattus norvegicus* (rat) (Figures 1 and 2; Tables S1A, B and C). The human IPCs, consisting of a total of 1748 pathways associated with 8624 genes, included 6224 genes mapped to 253 pathways within KEGG, 6085 genes mapped to 1272 pathways within Reactome and 2573 genes mapped to 223 pathways within NCI PID (Table 1). Below we discuss our observations on the integration of pathway data, their functional coherence and applications to the analysis of sample gene sets. Unless specified, all observations below correspond to the human pathway data.

**Table 1:** The number of human genes and pathways from different databases, which were consolidated into clusters of related pathways.

	KEGG	Reactome	NCI PID
**Pathways**	253	1272	223
**Genes**	6224	6085	2573

### Pathways within and Across Pathway Databases Share a Large Number of Genes in Common

Gene products may participate in multiple biological processes and pathways. Different pathway databases employ different approaches to compiling pathway information, and therefore may significantly differ in content; however, there remain some redundancies in pathway definitions within and across different databases. Therefore, we first examined gene overlaps between the pathways within each pathway database and across the three pathway databases. The gene overlap index (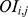) for a pair of pathways was determined by the ratio of the number of genes common to both the pathways to the number of genes within the smaller of the two pathways (see Methods).

A total of 242 pathways within KEGG, 202 pathways within NCI PID and 68 pathways within Reactome were examined for gene overlaps with each other in this manner (excluding pathways that were true subsets of one or more pathways). These included both intra-database (i.e., estimating gene overlaps between two pathways within a single database such as KEGG) and inter-database (i.e., estimating gene overlaps between two pathways from different databases) pathway comparisons. Amongst the intra-database pathway comparisons, we observed that 25 KEGG pathway pairs comprising 35 unique pathways (35 of 242; 14.4%) were remarkably similar (with 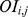 >0.8, i.e., the two pathways having 80% of their genes in common). Likewise, seven pathway pairs comprising eight unique pathways (8 of 202; 4%) and seven pathway pairs comprising 11 unique pathways (11 of 68; 16.1%) with 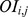 >0.8 were observed for NCI PID and Reactome databases, respectively (Table 2; Table S2). Amongst the inter-database pathway comparisons, for 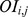 >0.8, we observed 29 KEGG-Reactome pathway pairs comprising 21 unique KEGG and 19 unique Reactome pathways: 12 KEGG-NCI-PID pathway pairs comprising 12 unique KEGG and seven unique NCI-PID pathways: and nine Reactome-NCI-PID pathway pairs comprising nine unique NCI-PID and six unique Reactome pathways (Table 2; Table S2).

**Table 2:** Pathway pairs within (intra-database) and across (intra-database) pathway datasets, with *OI_i,j_* >0.8.

	Intra-database			Inter-database		
	KEGG	Reactome	NCI PID	KEGG-Reactome	KEGG-NCI PID	Reactome-NCI
Pathway pairs	25	7	4	29	12	9
Unique pathways	35	11	8	21 KEGG, 19 Reactome	12 KEGG, 7 NCI PID	6 Reactome, 9 NCI PID

The pathway comparisons highlighted significant overlaps between apparently similar pathways within and across pathway databases. For instance, KEGG pathway hsa00970 “Aminoacyl-tRNA biosynthesis” shared a significant number of genes with Reactome pathway REACT_15482 “tRNA Aminoacylation” (with 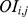 = 0.881); likewise, Reactome pathway REACT_75790 “Cytokine Signaling in Immune system” shared a significant number of genes with NCI PID pathway il5_pathway “IL5-mediated signaling events” (with 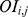 = 0.8571), among other examples. The above comparisons, however, also uncovered remarkable similarities between seemingly unrelated pathways; for instance, KEGG pathways hsa00190 “Oxidative phosphorylation” and hsa04966 “Collecting duct acid secretion” were found to have a significant number of genes in common (with 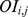 = 0.8519), suggesting that our approach towards pathway comparisons may provide insights into the possible cross-talks between varied biological processes (Table S2).

These observations suggest considerable overlaps among genes that were mapped to certain biological processes and pathway definitions within and across the three pathway repositories. These overlaps in information offer a useful means of consolidating large amounts of heterogeneous pathway data into a more manageable number of complimentary, broad-based and yet coherent biological themes, which is likely to contribute to a more streamlined functional analysis of genes and gene sets.

### Hierarchical Clustering of Pathways Based on Gene Overlap Indices

**Figure 3:**
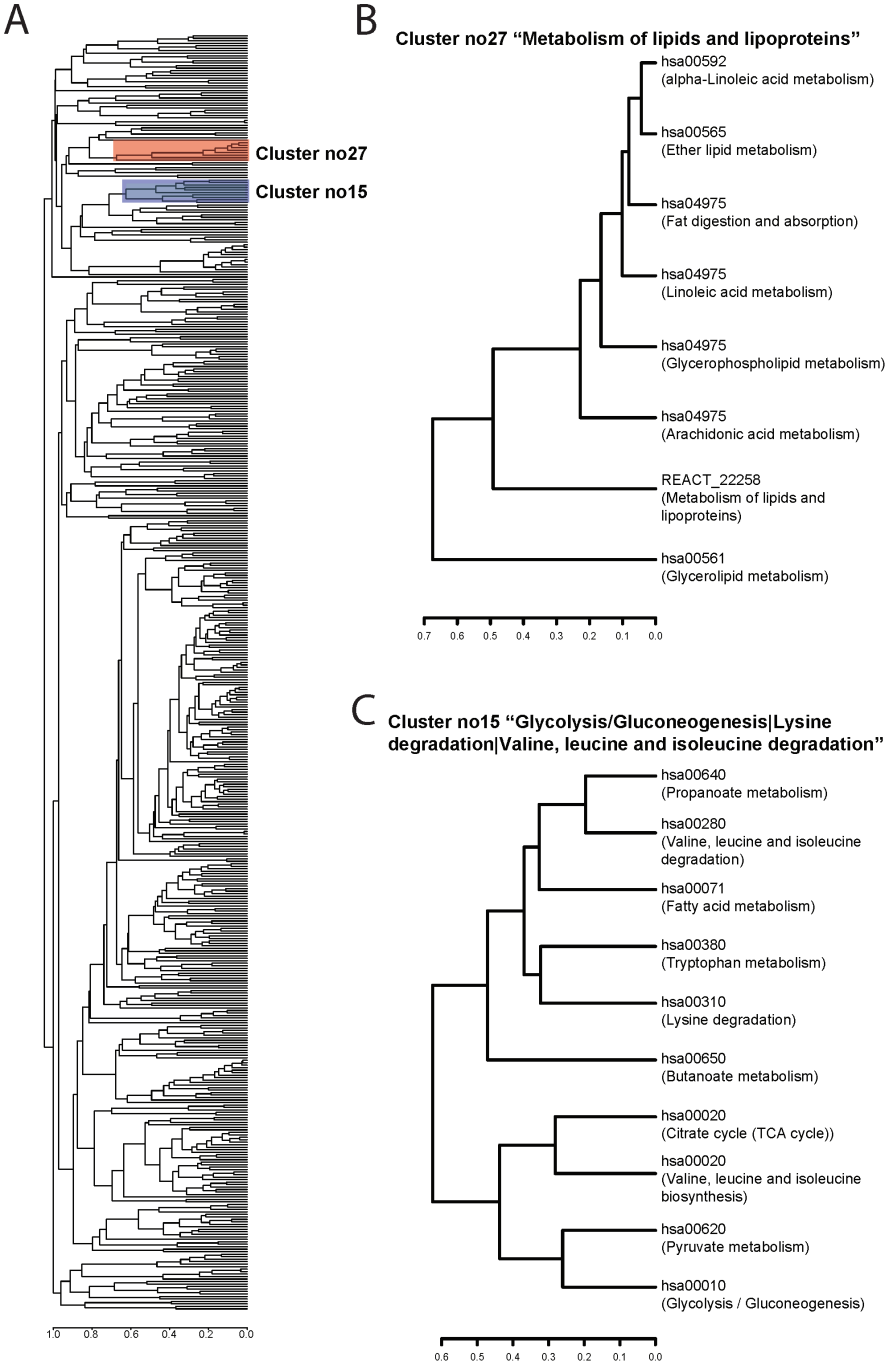
Hierarchical clusters of functionally related pathways based on gene overlap profiles. A) Dendrogram generated by using a matrix of gene overlap indices for all pairwise pathway comparisons. Specific pathway clusters no27 (red) and no15 (blue) are highlighted. B) Cluster no27 “Metabolism of lipids and lipoproteins” included eight pathways, all associated with lipid metabolism. C) Cluster no15 “Glycolysis/Gluconeogenesis | Lysine degradation | Valine, leucine and isoleucine degradation” consisted of ten pathways, most of which were associated with amino acid metabolism.

The gene overlap indices for all pairs of pathways were collated into a matrix, resulting in rows of overlap profiles. Based on these profiles, the pathways were then clustered to produce a dendrogram (Methods and Figure 3A).

Splicing the dendrogram at incrementally relaxed pairwise distance cutoffs generated a series of clusters; using cutoffs of 0.6, 0.65 and 0.7 yielded 105 (multi-member) clusters and 20 singletons, 84 clusters and 14 singletons and 67 clusters and 10 singletons, respectively.

After a visual inspection of the size distribution and the total number of multi-member clusters and singletons, we judged a cutoff of 0.7 to be optimal (likely to produce functionally congruent clusters while keeping the total number of clusters manageable) (Figure 3A). The pathway clusters thus generated varied in size from two pathways in clusters such as no18, no24 and no26 to 187 pathways in cluster no01 (Table S1A). The resulting clusters (hereafter referred to as IPCs) were further evaluated using a series of qualitative and quantitative measures to assess their functional congruency and biological relevance.

### Pathways within a Cluster Share a Higher Fraction of Genes than Those from Different Clusters

To investigate whether the gene overlap-based distance metric resulted in well separable clusters, we assessed the overall 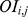 of pathways within and across the IPCs and compared the results with those from randomly generated pathway clusters.

The average intra-cluster 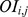 (0.175) was much higher than the average inter-cluster 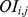 (0.022). The former value was significantly higher than the corresponding value from the randomised dendrograms (0.045±0.003) with a *p*-value of <0.01, as this value was greater than the maximum (0.053) from 100 simulation runs (see Methods).

The above observations suggest that our approach groups together pathways, which have a high fraction of genes in common and are therefore likely to be functionally related.

### Manual Inspection Revealed Selected Pathway Clusters Consisting of Functionally Related Pathways

A manual inspection of the pathway names within selected IPCs suggested that functionally similar pathways were grouped into clusters using our approach. For instance, cluster no27 “Metabolism of lipids and lipoproteins” included eight pathways (seven KEGG pathways and one Reactome pathway), all of which were associated with lipid metabolism (Figure 3B). Likewise, cluster no15 “Glycolysis/Gluconeogenesis | Lysine degradation | Valine, leucine and isoleucine degradation” included 10 pathways, most of which were associated with amino acid metabolism (Figure 3C). These observations suggest that pathway clusters generated by our approach are likely to include functionally related pathways and thereby likely to be biologically meaningful.

### Validation of the Functional and Biological Relevance of the Constructed Pathway Clusters

We further performed a series of quantitative assessments to examine whether the IPCs consisted of functionally related pathways and were biologically coherent and suitable for gene set analysis and target prioritisation. Below, we individually describe our observations on these evaluations.

#### Benchmarking pathway clusters against reference (KEGG pathway) sub-types

We employed *purity* and *edit distance* measures [13] to assess how well the KEGG pathways belonging to a particular reference sub-type (defined as the functional “categories” and “sub-categories” defined in the KEGG pathway database) were clustered together in non-singleton IPCs (see Methods for more details).

The *purity* scores for the IPCs when compared with the KEGG pathway categories and sub-categories were 0.48 and 0.22, respectively; these values were significantly higher than the average of the *purity* scores computed for randomised dendrograms (0.09 and 0.01, respectively) (Table 3) and were even higher than the maximum values (0.31 and 0.09, respectively) from 100 simulation runs; yielding a *p*-value of <0.01.

**Table 3:** *Purity* and *Edit distance* scores for the IPCs (*PD* = 0.7) when benchmarked against the KEGG pathway sub-types either at the top level (Main class) or the second level (Sub class) were much higher than those of the randomised dendrograms.

*PD*	*Purity*		*Edit distance*	
	Main class	Sub class	Main class	Sub class
0.7	0.48	0.22	93	137
Randomised dendrograms	0.09	0.01	158	249
0.6	0.54	0.28	123	153
0.65	0.52	0.29	109	149
0.75	0.48	0.24	88	134

*Purity* and *edit distance* scores at different *PD* cutoffs (0.6, 0.65 and 0.75) are included for comparison.

Likewise, the collective *edit distance* scores for all the IPCs were 93 and 137, respectively, which were much lower than the average of the collective *edit distance* scores computed for randomised dendrograms (158 and 249, respectively) (Table 3) and were even lower than the minimum values (133 and 233, respectively) from 100 simulation runs; yielding a *p*-value of <0.01.

The above observations suggest that IPCs described above correspond more closely to the reference sub-types as defined in the KEGG pathway database than randomised clusters and are thus, likely to represent biologically meaningful themes for functional annotation.

#### GO term semantic similarity-based evaluation of pathway clusters

Gene ontology (GO) annotations are one of the most useful and widely used means to estimate functional similarity between gene products. Semantic similarity is an approach to estimating the similarity or likeness between two terms of a given ontology (such as GO) [14]. In this study, the semantic similarity measure by Pesquita *et al*. [15] was extended to assess the functional similarity between a pair of (non-identical) pathways within an IPC or those found in different IPCs, thereby estimating the functional coherence of IPCs.

We first performed all-against-all pairwise pathway comparisons based on the GO term semantic similarities (GOSS) between their constituent genes (see Methods) and then we examined the overall functional similarity scores (*FS*) within and across IPCs (intra- and inter-cluster *FS*, respectively).

**Figure 4:**
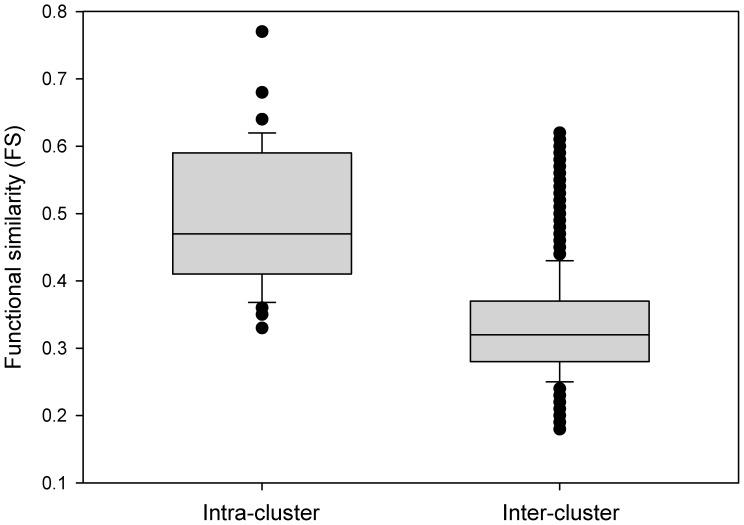
Functional similarity (*FS*) scores within a pathway cluster (Intra-cluster) were much higher than scores across pathway clusters (Inter-cluster).

The median *FS* within an IPC was significantly higher than the median *FS* across IPCs (0.47 and 0.32, respectively; *p* = 2.2×10^−16^ by the two-sided Mann-Whitney-Wilcoxon test, *W* = 114426.5) (Figure 4), thereby suggesting that the pathways within a cluster were functionally more closely related than the pathways in different clusters. We further compared *FS* observed within and across IPCs with those observed within randomised dendrograms. The median *FS* within an IPC was much higher than the average of median *FS* within a pathway cluster in the randomised dendrograms (0.47 and 0.33, respectively). Furthermore, the average *FS* within and across the clusters, 0.32 and 0.34, respectively, were statistically indistinguishable within randomised dendrograms.

Taken together, our observations suggested that IPCs were comprised of pathways, which shared an overall higher functional similarity with each other than with pathways from different clusters. Therefore, the pathway clusters were biologically meaningful and likely to represent coherent biological themes.

#### Gene set functional enrichment analysis

To assess the effectiveness of the IPCs in target prioritisation, we performed GSFE analysis on different sets of genes, which were known to be associated with hepatitis C virus (HCV) pathogenesis [16], [17], lung tumourigenesis in mice [18] and non-immune human diseases [19]. Below, we discuss the three case studies involving GFSE analysis using IPCs.

#### Case study I: Hepatitis C virus (HCV) pathogenesis

We examined four gene sets associated with HCV pathogenesis for enriched IPC associations. These included three gene sets comprising PPI networks constructed from differentially abundant proteins in transgenic mouse models of HCV pathogenesis (CoreTGvsWT, PA28γ^−/−^CoreTGvsWT and PA28γ^−/−^CoreTGvsCoreTG, respectively; see [16] for details) and a fourth gene set (NS5A infection network), which comprises genes associated with the cellular networks involved in interactions between HCV NS5A protein and human host factors [17]. Functional analysis of the CoreTGvsWT, PA28γ^−/−^CoreTGvsWT and PA28γ^−/−^CoreTGvsCoreTG gene sets highlighted enriched associations with 28, 28 and 29, IPCs respectively. These figures were much lower than the number of individually enriched KEGG (91, 99 and 98) Reactome (416, 372 and 433) and NCI PID (134, 120 and 113) pathways associated with the above gene sets (Table S3A).

Among specific examples, the IPC no64 “SNARE interactions in vesicular transport” was highly enriched in all of the first three gene sets (*p* = 0.01, *p* = 6.25×10^−5^ and *p* = 2.86×10^−4^, respectively) (Table S3A). In our previous analysis [16], after inspecting a much larger number of enriched pathways (as shown above), including those with relatively weak *p*-values, we demonstrated experimentally the involvement of vesicular transport proteins in HCV lifecycle. This finding would have been achieved more easily with the IPC analysis.

The NS5A infection network was associated with 25 enriched IPCs; this figure was much lower than the number of individually enriched KEGG (98), Reactome (488) and NCI PID (119) pathways associated with the NS5A infection network. The enriched IPCs included no23 “Endocytosis | Tight Junction” (*p* = 6.84×10^−18^) (Table S3A). Cluster no23 includes genes and pathways associated with cell adhesion and communication and cellular transport, some components of which had been strongly implicated in facilitating HCV lifecycle and tumourigenesis in HCV-induced hepatocellular carcinoma (HCC) [17]. In general, the enriched IPCs included all the biological themes that we had identified previously from a much larger list of relevant pathways and subsequently validated experimentally.

In some enriched IPCs, genes in the original gene set were mapped to two or more pathways, which were not enriched individually. For instance, within the PA28γ^−/−^CoreTGvsCoreTG network, two autophagy associated factors GABARAPL1 and GABARAPL2 were mapped to enriched IPCs no011 “Cytokine Signaling in Immune system| Cytokine-cytokine receptor interaction | Herpes simplex infection | Tuberculosis” and no012 “GPCR ligand binding | Neuronal System| Neuroactive ligand-receptor interaction” (*p* = 2.61×10^−28^ and *p* = 2.18×10^−8^, respectively). These two genes would not have been identified by the standard pathway analysis, because they were mapped to two KEGG pathways hsa04140 “Regulation of autophagy” and hsa04727 “GABAergic synapse”, which were components of no011 and no012, respectively and neither of which showed significant association with the original gene set (*p* = 0.7395 and *p* = 0.0867, respectively) (Table S3B). Recent studies have implicated autophagy response to HCV-induced endoplasmic reticulum stress in impairing Type I interferon production in HCV infection [20]. Therefore the analysis using IPCs was able to identify a novel biological theme not identifiable by previous methods.

#### Case study II: Lung tumourigenesis

We also performed a functional analysis of genes involved in the function of transcription factor Stat3 in carcinogen-induced lung tumourigenesis in mice [18]. Two gene sets examined (Stat3-upreg and Stat3-downreg, respectively) corresponded to PPI networks constructed from differentially expressed genes in Stat3 knockout mice. The Stat3-upreg and Stat3-downreg gene sets were associated with seven and six enriched IPCs, respectively. Among specific examples, Stat3-upreg was mapped to enriched pathway cluster mmu045 “TGF-beta signalling pathway” (*p* = 0.003) and Stat3-downreg was associated with an enriched pathway cluster mmu021 “Rheumatoid arthiritis” (*p* = 0.019) (Table S4A). Furthermore, within the Stat3-upreg gene set, complement activation-associated factor Cfh was mapped to enriched IPC no005 “Hemostasis | Disease | Adaptive Immune System | Pathways in cancer | HTLV-I infection | MAPK signaling pathway” (*p* = 4.73×10^−7^). The above association would not have been identified by the standard pathway analysis, because Cfh was mapped to three Reactome pathways REACT_86987 “Innate Immune System”, REACT_144679 “Regulation of Complement cascade” and REACT_103920 “Complement cascade”, which were components of no005 but individually, none of the three pathways showed significant association with the original gene set (*p* = 0.3206, *p* = 0.3430 and *p* = 0.6079, respectively) (Table S4B). Our observations appear to be consistent with previous studies, which have shown that the human orthologue of mouse Cfh is associated with the early stages of lung tumourigenesis [21], [22]. These results demonstrate the relative ease of identifying enriched biological processes previously shown to play critical roles in Stat3-dependent carcinogen-induced lung tumourigenesis [18] and the ability of our approach to identify a novel biological theme not identifiable by previous methods.

#### Case study III: Non-immune human diseases

An IPC representing a general biological theme of the immune system (no1; Adaptive Immune System | Hemostasis | Developmental Biology | Pathways in cancer | Innate Immune System) was enriched in all of the gene sets above. To confirm that this result was not an artefact of the clustering method, we performed a functional analysis of gene sets associated with non-immune human diseases, Atherosclerosis, Hypercholesterolemia and Pancreatitis. Our enrichment analysis revealed an enrichment of five IPCs for each of the three gene sets, respectively. These figures were much lower than the number of individual enriched KEGG (31, 18 and 48) Reactome (51, 23 and 43) and NCI PID (9, 1 and 13) pathways associated with the above gene sets (Table S5).

Amongst the most significant associations, IPC no027 “Metabolism of lipids and lipoproteins” was associated with the Hypercholesterolemia gene set (*p* = 1.13×10^−21^) (Table S5), which is consistent with the perturbations in lipid metabolism in this disease [23]; enriched IPC no010 “Dilated cardiomyopathy | ECM-receptor interaction | Integrin cell surface interactions” and no027 “Metabolism of lipids and lipoproteins” were associated with the Atherosclerosis gene set (*p* = 6.96×10^−7^ and *p* = 0.008, respectively) (Table S5), which is consistent with the pathology of the cardiovascular disease [24]; IPC no014 “Biological oxidations | Metabolism of xenobiotics by cytochrome P450” and no040 “Glutathione metabolism” were associated with the Pancreatitis gene set (*p* = 7.66×10^−8^ and *p* = 1.58×10^−5^, respectively), which is consistent with the xenobiotic stress and glutathione depletion associated with chronic pancreatitis [25].

The above examples suggest that our IPCs were able to provide a relatively quick, manageable and meaningful approximation of biological themes associated with diverse gene sets.

### Data Visualisation and Accessibility

A web interface, tightly connected to TargetMine, was developed for visualising the IPCs and performing GSFE (http://targetmine.nibio.go.jp/pathclust/). It allows a user to upload a list of candidate genes (such as a list of differentially expressed genes, or a set of genes whose protein products interact with a given protein) to TargetMine and create a gene list. The user can then retrieve enriched IPCs and examine their pathway and gene content. Further analysis of these genes and pathways may be performed using TargetMine with its query builder or pre-defined templates.

**Figure 5:**
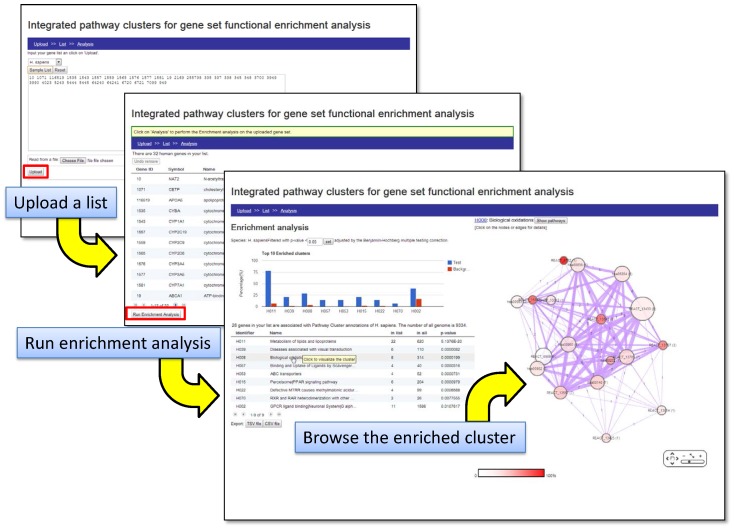
The online user interface allows the users to query and visualise integrated pathway clusters and perform GSFE analysis with the supplied list of genes.

Each IPC is visualised as a network graph, with the nodes representing the pathways and the edges representing gene overlaps between them. The size of each pathway node reflects the number of genes within that pathway and the thickness of the edges connecting individual pathways reflect the extent of gene overlaps between the connected pathway nodes. A mouse over function allows the user to highlight individual pathways within a cluster; the gene content of each pathway may also be displayed with mouse clicks (Figure 5).

### Comparison with Related Resources

The availability of the IPCs within a data warehouse environment makes our approach different from most other integrated pathway repositories such as IntPath [6], IPAD [5], PathwayAPI [1] and Pathway Distiller [7], as well as more general gene function annotation tools such as DAVID [4]. None of these tools provide seamless links with biological data types, other than the integrated biological themes available within these repositories. In contrast, our data model for the IPCs enables the users to link up these functional associations with diverse biological data types stored in TargetMine, such as disease phenotypes, protein structural domains and drug–target associations.

Some of these integrated pathway repositories employ more complex approaches than ours and/or include additional pathway and biological datatypes to infer integrated pathway clusters. Our method is simple and fast and IPCs can be updated automatically. It can also be extended to a larger number of pathway databases or even to other biological data types such as GO annotations.

Among the existing integrated repositories, the Human Pathway Database (HPD) is the closest to our approach in that it integrates pathway data from KEGG, Reactome, NCI PID and BioCarta based on gene/protein overlaps and provides a standalone web interface to query large gene sets for human pathways within a data warehouse [26]. Its data warehouse framework is a less comprehensive system than TargetMine and HPD only considers the extent of gene/protein overlap between pathways to estimate pathway similarity, whereas our approach considers not only gene overlaps but also the similarity of the gene overlap profiles.

hiPathDB adopts a full integration approach where individual pathways are consolidated into a unified derivative superpathway based on shared components. This method provides a holistic and a concise view of biological processes including cross talks between different signalling pathways, but it also results in a loss of information at the molecular level [27]. Our IPCs are designed to complement the existing functional annotations and our data model allows the users to revisit the underlying gene-pathway associations in their original form.

Other differences between our IPCs and the clusters (groups of functionally related genes) defined in the popular DAVID gene functional classification tool include 1) automatically assigned informative names for the IPCs (in contrast to the DAVID clusters with no representative names), and 2) visualisation of IPCs as network graphs to allow the users to examine connections and relationships between constituent pathways.

## Conclusions

We describe our approach to integrating pathway information from public repositories based on shared gene content into functionally coherent pathway clusters. The resultant IPCs provided a convenient way to identify broad functional categories relevant to the biological phenomenon under study and thereby enabled swift candidate gene prioritisation. Since our approach relies only on gene overlap between pathways, its inherent flexibility ensures that data from additional pathway repositories (and even non-pathway gene sets) can be readily accommodated to expand the content and coverage of the IPCs.

We assessed the quality of the IPCs using multiple independent measures, including the agreement with the reference sub-types defined in the KEGG database and intra- and inter-cluster semantic similarity scores. With the help of these measures, we established that the IPCs were functionally coherent and biologically meaningful. We further demonstrated the ease of employing the IPCs to analyse large gene sets extracted from the literature.

Our fully automated approach has been integrated into the TargetMine data warehouse and enhanced its ability to investigate complex biological systems for better target discovery. It has also enabled seamless updates of the IPCs synchronised with TargetMine updates, which are scheduled every month in general.

## Materials and Methods

### Pathway Data

An overview of our approach to overcoming the challenges encountered in integrating diverse pathway data is shown in Figure 2. In the present analysis, pathway associations for the genes within the human, mouse and rat genomes were extracted from KEGG (retrieved on 16/06/2012), Reactome (release date 26/06/2012) and NCI-Nature curated Pathway interaction database (retrieved on 05/04/2012) repositories. The non-IEA (Inferred from Electronic Annotation) GO annotations for the corresponding genes above were retrieved using the TargetMine data warehouse [8]. Some pathways are broadly defined and include many genes (for example, KEGG pathway “Metabolic pathways”). Since these pathways are uninformative for gene prioritisation purposes, we set an arbitrary cut-off of 700 and excluded eight such pathways with more than this number of genes from the subsequent analysis.

### Estimating Agreements Across Pathways Based on Gene Composition

Next, we examined the agreement between different pathways within and across pathway repositories based on the overlaps of their gene composition. For each pathway 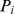 in the dataset (where *i* = 1,…,*N* and *N* is the total number of pathways), let 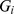 be the set of genes in the pathway. For a pair of pathways, 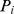 and 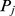, 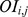 was defined as
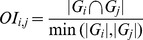
where 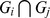 is the set of genes shared by 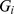 and 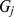 and 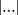 is the number of genes in the set [28]. An 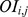 of 1 indicates that either the two pathways are identical in size and gene composition or that one pathway is a true subset of the other. To simplify the computation, pathways that were true subsets of larger pathways (“fully contained pathways”) were excluded from the subsequent pairwise comparisons (but they were reintroduced into the final clustering results, as will be described later). Likewise, an 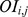 of 0 indicates that the two pathways have no genes in common.

### Pathway Clustering Based on Gene Overlap Indices

The collective gene overlap indices for each pathway were then collated to generate corresponding gene overlap profiles. We defined each row 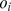 of the matrix 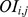 as the gene overlap profile of pathway 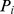. The Pearson correlation coefficient *R* was calculated for each pair of gene overlap profiles (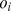 and 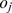) and transformed into pairwise distances *PD* as 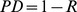. With this distance metric, average-linkage clustering was performed using the hclust function of the R statistical package (www.r-project.org). The dendrogram was partitioned at incremental *PD* cutoffs and the resulting clusters of related pathways were manually examined to select the most suitable cutoff (see Results).

Once the pathway clusters were established, the fully contained pathways were reintroduced into the clusters that included their “parent” pathways. (If a fully contained pathway had more than one parent and these parent pathways belonged to different clusters, the fully contained pathway was assigned to all these clusters.).

### Pathway Cluster Naming

To provide the pathway clusters with informative labels, we examined the gene composition of each pathway cluster and identified the pathways that collectively contributed ≥50% of the genes within a cluster. Their entry names in the original database were then assigned to the corresponding cluster. (In case of two or more pathways contributing ≥50% genes within a cluster, their entry names were concatenated to assign cluster names.).

### Assessing the Functional Homogeneity within the Pathway Clusters Based on KEGG Pathway Sub-types

We adapted the *purity* and *edit distance* measures as defined by Brown *et al*. [13] to assess the efficacy of the pathway clustering approach. These scores were used to assess the consistency between our pathway clusters and reference sub-types of pathways as defined in KEGG; KEGG classifies its pathways into “categories” (at the top level such as Metabolism, Cellular Processes and Human Diseases) and “sub-categories” (at the second level such as Energy metabolism, Cell growth and death and Immune system). Only KEGG pathways within a cluster were evaluated in this manner. Clusters containing only one pathway (singletons) were excluded from the following analysis.

In this study, *purity* was defined as the fraction of the constructed pathway clusters that consisted entirely of KEGG pathways belonging to a single reference sub-type (“category” or “sub-category”) and were therefore, “functionally homogenous”. Here, *purity* reflects the efficacy of our approach in resolving the pathway clusters into functional categories corresponding to KEGG pathway sub-types; a *purity* score of 1 indicates that all KEGG pathways within each pathway cluster were mapped to a single KEGG sub-type, whereas a *purity* score of 0 indicates that none of the pathway clusters were functionally homogenous.

Likewise, in this study, *edit distance* was computed as the minimal number of split and/or merge operations, which were required to transform individual pathway clusters into a KEGG pathway sub-type. For instance, if the pathways corresponding to the KEGG sub-type “Immune system” are distributed across two clusters, each containing other KEGG pathways, two split and one merge operations would be sufficient to transform the two clusters into a single cluster containing all pathways within the KEGG “Immune system” sub-type. The *edit distance* for this process would be 3.

To assess the statistical significance of these measures, 100 randomised dendrograms were generated by shuffling the pathways across the clusters in a manner such that the number of pathway clusters and the number of pathways within a given cluster were preserved. The randomised dendrograms were used to create 100 random sets of pathway clusters.

We defined the *p*-value of the significance of these two observations (*purity* and *edit distance*) using the fraction of the *purity* and *edit distance* scores amongst the randomised conditions that was greater than the actual *purity* and *edit distance* score of the constructed pathway clusters.

### Functional Similarities of Pathways and Pathway Clusters

We extended the GOSS [14] defined between a pair of genes to those between a pair of pathways and used this measure to assess the functional similarities within a pathway cluster (intra-cluster coherence) or between pathway clusters (inter-cluster separation). First, the algorithm of Wang *et al*. [29] was employed via an in-house Scala/Java implementation to estimate the GOSS between a pair of genes. This method takes into account both the properties of the annotated GO terms including their parent and child terms and the types of relationships between them (such as “is_a” and “part_of”, which are assigned semantic contribution weights of 0.8 and 0.6, respectively).

Next, we defined functional similarity (*FS*) between a pair of pathways, 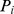 and 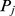, as fellows. *FS* may be naturally defined by calculating all possible pairwise GOSS values between genes in 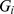 and 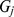. However, such a measure would simply reflect the amount of overlap between 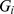 and 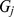, which was already taken into account in our clustering algorithm. Since we wished to assess the quality of our pathway clusters based on non-trivial functional similarities between the constituent pathways, we needed to remove contributions from the overlapping genes.

To achieve this goal, in considering a pair 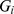 and 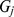, let 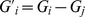, i.e., a set of genes in 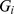 but not in 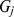. Similarly, let 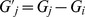. By adopting a best-match average approach analogous to that of Pesquita *et al*. [15], the best match functional similarity score 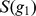 for each 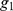 in 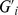 was defined as
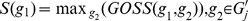
where the maximum was taken over all 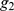 in 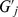. The GOSS for a pair of genes, 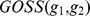, was defined by [15] as:



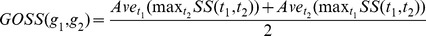
where 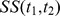 is the GO semantic similarity between two terms 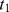 and 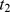, 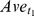 means taking the average over all the terms 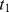 that were assigned to gene 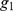 and the maximum was taken over all the terms 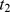 that were assigned to gene 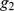. In other words, this measure represents the average similarity between each term assigned to 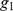 and its most similar term among those assigned to 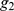, averaged with its reciprocal to obtain a symmetric score.

Finally, by using 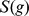 above, the functional similarity for a pathway pair, 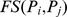, was defined as:
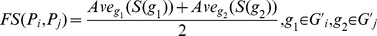
where 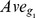 means taking the average over all 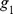 in 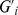.


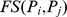
 was computed for all pathway pairs within a cluster (intra-cluster) and for all pathway pairs across different clusters (inter-cluster).

To assess the statistical significance of the intra- and inter-cluster *FS* scores, 100 randomised dendrograms were generated (as described in the previous section) and used as controls. The intra- and inter-cluster 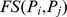 was computed for each randomised dendrogram and these collective scores were then compared with the intra- and inter-cluster 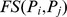 of the constructed pathway clusters.

### Functional Enrichment Analysis of Gene Sets using Pathway Clusters

Functional enrichment analysis was performed on human and mouse gene sets extracted from the literature. These included gene sets associated with HCV pathogenesis [16], lung tumourigenesis in mice [18] and non-immune disease-related gene sets [19]. The above gene sets were mapped to the IPCs and the enrichment of specific functional categories was estimated by performing Fischer’s exact test. The inferred *p*-values were further adjusted for multiple test correction to control the false discovery rate using the Benjamini and Hochberg procedure [30], [31] and the annotations/pathways were considered significant if the adjusted *p*≤0.05.

### Visualisation and Web Interface

The visual representation of the pathway clusters was implemented with JavaScript libraries including jQuery and Cytoscape Web.

## Supporting Information

Table S1 Integrated pathway clusters A) Human. B) Mouse. C) Rat.
(XLSX)

Table S2 Pathway pairs which share *OI_i,j_* ≥0.8.
(XLSX)

Table S3 A) Enriched IPC associations for the HCV pathogenesis-associated datasets. B) Enriched IPCs associated with genes within the HCV pathogenesis-associated datasets, which were mapped to one or more non-enriched pathways.
(XLSX)

Table S4 A) Enriched IPC associations for the Lung tumourigenesis-associated datasets. B) Enriched IPCs associated with genes within the Lung tumourigenesis-associated datasets, which were mapped to one or more non-enriched pathways.
(XLSX)

Table S5 Enriched IPCs associated with genes within the Non-immune human diseases-associated datasets, which were mapped to one or more non-enriched pathways.
(XLSX)
